# The molecular mechanisms of quality difference for Alpine Qingming green tea and Guyu green tea by integrating multi-omics

**DOI:** 10.3389/fnut.2022.1079325

**Published:** 2023-01-06

**Authors:** Hongshi Xiao, Jie Yong, Yijie Xie, Haiyan Zhou

**Affiliations:** ^1^College of Bioscience and Biotechnology, Hunan Agricultural University, Changsha, China; ^2^Agricultural and Rural Bureau of Hefeng County, Hefeng, China

**Keywords:** Hefeng tea, transcriptomics, *Camellia sinensis*, endophytic bacteria, green tea

## Abstract

**Introduction:**

Harvest time represents one of the crucial factors concerning the quality of alpine green tea. At present, the mechanisms of the tea quality changing with harvest time have been unrevealed.

**Methods:**

In the current study, fresh tea leaves (qmlc and gylc) and processed leaves (qmgc and gygc) picked during Qingming Festival and Guyu Festival were analyzed by means of sensory evaluation, metabolomics, transcriptomic analysis, and high-throughput sequencing, as well as their endophytic bacteria (qm16s and gy16s).

**Results:**

The results indicated qmgc possessed higher sensory quality than gygc which reflected from higher relative contents of amino acids, and soluble sugars but lower relative contents of catechins, theaflavins, and flavonols. These differential metabolites created features of light green color, prominent freshness, sweet aftertaste, and mild bitterness for qmgc.

**Discussion:**

Flavone and flavonol biosynthesis and phenylalanine metabolism were uncovered as the key pathways to differentiate the quality of qmgc and gygc. Endophytic bacteria in leaves further influence the quality by regulating the growth of tea trees and enhancing their disease resistance. Our findings threw some new clues on the tea leaves picking to pursue the balance when facing the conflicts of product quality and economic benefits.

## Introduction

Green tea is a well-known beverage native with health benefits and pleasant taste (1). The tea plant [*Camellia sinensis* (L.) O. Kuntze] grows widely in tropical and subtropical regions around the world, primarily in China, Japan, Argentina, Vietnam, India, and Kenya (2). With the growth of tea tree cultivation areas, a surge in processing products and exploitation is expected in the near future. In order to cater to the healthy food market demand, it is necessary to develop tea products with local characteristics and high quality.

Hefeng green tea is regarded as the specialty of Enshi (Hubei Province, China), the selenium capital of the world. This particular tea is known for its excellent taste, color, and aroma, attributed to the ecological environment which is far from industrial pollution and has selenium-rich soil. Moreover, Hefeng tea also possesses some properties including anti-cancer, anti-aging, immunity, and fertility-enhancing.

The tea quality is positively correlated with the altitude of cultivation (3), and largely depends on the content of secondary metabolites such as flavonoids, phenolic acids, and alkaloids (4). Tea polyphenols, citric acid, theanine, and sucrose normally increase with altitude, while proanthocyanidin C1, theanine B, and catechins show a decrease with altitude (3). The high-altitude slows down the growth rate of Hefeng tea relative to other varieties in the same latitude, and definitely leads to delayed marketing dates and a competitive disadvantage. Moreover, some traditional planting patterns without pruning, pesticides, and fertilizers also further delayed its market timing, while preserving the original flavor of Hefeng green tea. All of these traditional methods enhance the quality of green tea. For instance, shade directly leads to the down-regulation of epigallocatechin gallate, catechin gallate, anthocyanins, and proanthocyanidins, resulting in marked enhancement in tea quality (5). It is also noteworthy that green tea processed from unpruned tea plants has better aroma and taste (6).

Furthermore, some researchers have claimed that the harvest time exerts a significant impact on the tea quality. One previous study indicated that in early spring tea leaves, the concentrations of amino acids (L-glutamine and L-tryptophan), (S)-(-)-limonene, catechins, and flavonol/flavone glycosides were higher, while the concentrations of proanthocyanidins (proanthocyanidin A1, protofibronectin A1, and protofibronectin A2 3′-gallate) were diminished compared to the control. There are also vast differences in the metabolic profiles of young tea leaves in early spring and late spring, which can be attributed to certain close-related biosynthetic pathways like flavonoids, phenylpropanoids, flavonoids, and flavonols, phenylalanine, tyrosine, and tryptophan (7). In one experiment (8), all of the early, middle, and late spring green teas at low altitudes were analyzed by gas chromatography-time-of-flight mass spectrometry, the results of which revealed that with decreasing concentration of amino acids, there was a strong enhancement in the concentration of carbohydrates, flavonoids, and their glycosides in the late spring season, which was feedbacked from the sensory quality of the tea leaves made.

In Hefeng (Enshi), fresh tea leaves picked at the Qingming Festival are generally used to make high-grade green tea, while fresh tea leaves picked at the Guyu Festival are generally used to make ordinary green tea. Both the Qingming Festival and the Guyu Festival belong to the 24 solar terms in the Chinese lunar calendar. The Qingming Festival falls around the 5th of April each year and 15 days earlier than Guyu Festival. This 15-day waves down the price of green tea sensitive.

In brief, it is necessary to probe into the relationship between the harvest time and the variation of key differential metabolites relative to the quality of alpine green tea. The Wufengshan green tea grown in high-attitude mountains was selected as the focus of the current study. Its fresh leaves collected at the Qingming Festival and Guyu Festival were processed into commercial products through a traditional process. To explain the changes in key metabolic pathways with harvest time, metabolomics was utilized to detect differential metabolites (DEMs) in the samples, and transcriptomics was adopted to identify differentially expressed genes (DEGs). Meanwhile, the effect of symbiotic bacteria on the quality of alpine green tea was discussed by high-throughput sequencing technology. Our study aims to objectively evaluate the quality difference between Qingming green tea and Guyu green tea at the molecular level to shed new light on the optimization of tea processing technology.

## Materials and methods

### Plant materials

Firstly, six Wufengshan green tea plants with 15-year-olds were divided into two groups. Fresh leaves were collected from Wufengshan tea plantation (110.091363°E, 29.845475°N, Hefeng, Hubei Province, China) at an altitude of 1,450 m on 5 April 2022 (11:00 a.m.) and 20 April 2022 (11:00 a.m.), respectively. Samples collected under aseptic conditions were wrapped in tin foil and labeled. A portion of the samples was rapidly frozen with liquid nitrogen for 15 min and stored at −80°C for subsequent experiments. The remaining portion of the samples was prepared directly to make green tea according to the local traditional process as follows: spreading → killing → kneading → initial drying → shaping and extracting hairs → full drying → aroma. One part of processed samples was stored at 4°C for sensory evaluation, and the other part was stored at −80°C for subsequent experiments.

### Sensory evaluation

Sensory evaluation of Qingming green tea (qmgc) and Gu Yu green tea (gygc) was performed independently by five professional tasters with methodological reference (9). The score was 100 points as follows: appearance color (25%), brew color (10%), aroma (25%), taste (30%), and infused leaves (10%).

### Metabolomic analysis

Twenty milligrams of freeze-dried samples were added to 1,000 μl of extract (containing 70% methanol and isotope-labeled internal standard mixture), ground at 35 Hz for 4 min, and sonicated in an ice-water bath for 5 min. The samples were subsequently incubated at −40°C for 1 h and then centrifuged at 4°C for 15 min at 12,000 rpm. The supernatant and QC samples (an equal mixture of all samples) were collected for metabolomics assay.

The chromatographic separation of the target compounds was performed using a Vanquish (Thermo Fisher Scientific) ultra-performance liquid chromatograph and a Waters ACQUITY UPLC HSS T3 (2.1 mm × 100 mm, 1.8 μm) liquid chromatographic column. A phase of the liquid chromatography was aqueous (containing 5 mmol/L ammonium acetate and 5 mmol/L acetic acid), and B phase was acetonitrile. Sample tray temperature: 4°C, injection volume: 2 μl. An Orbitrap Exploris 120 mass spectrometer (Xcalibur, version: 4.4, Thermo Fisher Scientific) was adopted for mass spectrometry data acquisition. The raw data were processed by peak identification, peak extraction, peak alignment, and integration and then matched with BiotreeDB (V2.1) secondary mass spectrometry database for substance annotation.

### Transcriptomic analysis

Total RNA content was extracted from fresh tea samples using the Trizol reagent (Thermo Fisher Scientific, 15596018). The obtained RNA quantity and purity were analyzed with Bioanalyzer 2100 and RNA 6000 Nano LabChip Kit (Agilent, CA, USA, 5067-1511), respectively. Next, the mRNA was purified using Dynabeads Oligo (Thermo Fisher Scientific, CA, USA). Library construction was subsequently performed with the VAHTS™ Stranded mRNA-seq Library Prep Kit for Illumina^®^ (Nanjing Novaseq NR601-01). Afterward, the cDNA libraries were sequenced using the Illumina Novaseq™ 6000 system.

The raw images obtained from high-throughput sequencing were transformed into raw sequences by means of base calling analysis, and the clean data were collected by filtering out the unqualified sequences with the Cutadapt tool and pre-processed. The pre-processed valid data was then compared with the reference genome (“shuchazao”) using Hisat2. Based on the results of Hisat2 alignment, transcripts were reconstructed using the StringTie transcript assembler, followed by calculation of the expression levels of all genes in each sample. DESeq2 software was adopted for differential gene expression analysis between the two groups.

Transcripts of genes related to flavonoid biosynthesis were randomly selected for qRT-PCR validation with GAPDH serving as the internal reference gene. qRT-PCR reaction parameters were as follows: 95°C for 10 min, 94°C for 10 s, 58°C for 15 s, for a total of 45 cycles. Fluorescence intensity was detected using the LightCycler 480 system (Roche, Sussex, UK), and then the relative expression values of genes were calculated.

### Diversity analysis of endophytic bacteria

Genomic DNA content was extracted using the PowerSoil^®^ DNA isolation kit under aseptic conditions, and the quality of DNA extraction was determined by 1% agarose gel electrophoresis. PCR amplification was performed using primers specific for the V7-V9 region of the 16S rRNA gene: 799F-1193R 5′-AACMGGA TTAGATACCCKG-3′ and 5′-ACGTCATCCCCACCTTCC-3′. The PCR reaction conditions were as follows: 95°C for 5 min; 95°C for 1 min, 50°C for 1 min, 72°C for 1 min, for a total of 35 cycles; 72°C for 7 min. The amplification products were detected using 1% agarose gel electrophoresis and sent to the Illumina NovaSeq platform for sequencing.

Following sequencing, the data were spliced, quality-controlled, and chimera filtered by overlap to obtain high-quality clean data. Single-base precision representative sequences were obtained using the DADA2 algorithm, and then ASVs (Amplicon Sequence Variants) were adopted to construct class OTUs (Operational Taxonomic Units), and to obtain the final ASV feature table as well as the feature sequences for further diversity analysis, species taxonomic annotation, and difference analysis, etc.

### Data analysis and statistics

Statistical analyses were processed using the SPSS software. Diagrams were drawn by OriginPro 2018 and Adobe Illustrator CC 2019.

## Results and discussion

### Differences between qmlc and gylc products from sensory perception

**FIGURE 1:**
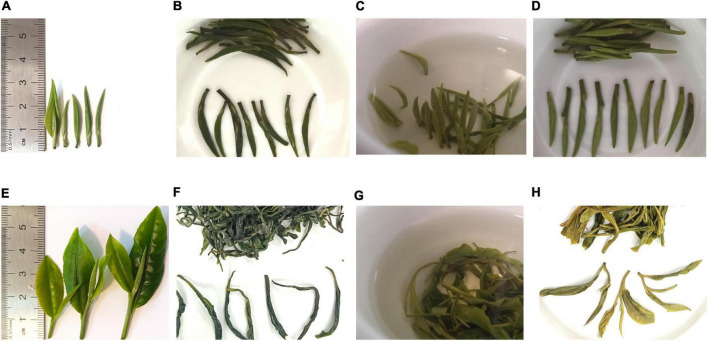
Sensory evaluation of Qingming tea and Guyu tea. **(A)** Appearance of qmlc. **(B)** Appearance of qmgc. **(C)** Brew color of qmgc. **(D)** Infused leaves of qmgc. **(E)** Appearance of gylc. **(F)** Appearance of gygc. **(G)** Brew color of gygc. **(H)** Infused leaves of gygc.

As illustrated in Figure 1 and Supplementary Table 1, there were obvious phenological differences between the two kinds of green tea. Most of the qmlc leaves presented with white fluffy buds or a small amount of one leaf and one bud in the length of 30 mm, otherwise the green leaves were slightly spreading or not spreading. Meanwhile, the vast majority of gylc leaves had two leaves and one bud or a small amount of one leaf and one bud in the length of 60 mm, and the leaves appeared translucent yellow-green coloration in sunlight.

Furthermore, in regard to appearance color, aroma, taste, brew color, and infused leaves of gygc and qmgc, it was found that qmgc was of better quality with a higher total score, especially in appearance color. In addition, qmgc retained the straight shape and was further covered by silvery-white hairs with a light green shiny color. Existing studies indicate that these hairs contribute to the defense of the tea plant, the flavor, and nutritional quality of leaves (10). Gygc possesses a curved shape, and is covered by a few silvery white hairs with a dark green oily color. Meanwhile, in regard to the aroma, gygc was found to be better than qmgc. qmgc was dominated by soft floral and fruity aromas, while gygc exhibited a strong and persistent honeysuckle aroma and chestnut aroma. The latter differences are important as aroma is regarded as one of the most crucial factors in evaluating tea quality, and high-quality green tea often emits clean or chestnut aromas (11). Moreover, in terms of taste, qmgc was pure, tasty, and sweet with a lighter flavor. On the other hand, gygc had a strong flavor with sweetness, but also more pronounced bitterness. Further in regard to brew color, qmgc was bright green, while gygc was bright greenish-yellow. On the infused leaves, qmgc could stand upright in the glass for a short time and spread naturally after fully absorbing water without producing crumbs. Although, gygc leaves could not fully spread after absorbing water and some leaves were mutilated, while some exhibited yellowish coloration. The above findings indicated that both green teas could meet the standard of Hefeng green tea production,^1^ and qmlc was more suitable for processing as high-grade handmade green tea.

### Differences of secondary metabolism products from metabolomics analysis

**FIGURE 2:**
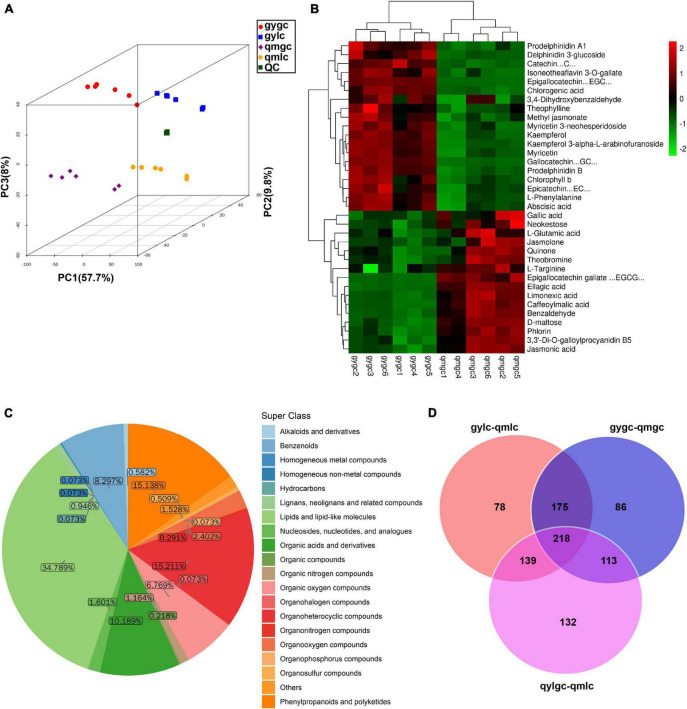
The metabolomic analysis of tea samples. **(A)** Score scatter plot for PCA model total with QC. **(B)** Key DEMs determining the difference in quality between gygc and qmgc. **(C)** Metabolites detected in all tea samples. **(D)** Venn diagram of metabolite statistics for differences between tea sample groups.

**FIGURE 3:**
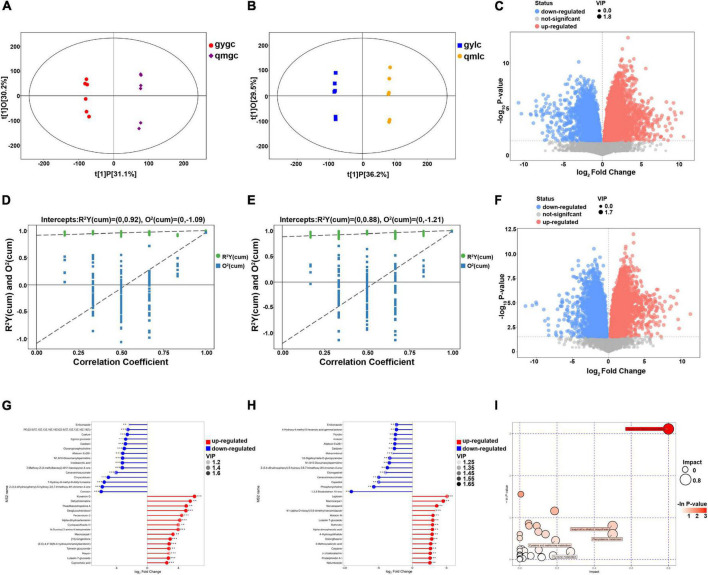
Metabolomic analysis of gylc and qmlc. **(A)** Score scatter plot of OPLS-DA model for group gygc vs. qmgc. **(B)** Score scatter plot of OPLS-DA model for group gylc vs. qmlc. **(C)** Volcano plot for group gylc vs. qmlc. **(D)** Permutation plot test of OPLS-DA model for group gygc vs. qmgc. **(E)** Permutation plot test of OPLS-DA model for group gylc vs. qmlc. **(F)** Volcano plot for group gylc vs. qmlc. **(G)** Matchstick analysis for group gygc vs. qmgc. **(H)** Matchstick analysis for group gylc vs. qmlc. **(I)** Pathway analysis for group gylc vs. qmlc.

Metabolites serve as reflectors of the physiological state of tea plants, and DEMs are the direct cause of quality differences between qmgc and gygc. A prior study observed 20,971 peaks from the data of the Q Exactive LC-MS/MS platform in positive ion mode (POS). PCA analysis in our study (Figure 2A) illustrated that all samples fell into the 95% confidence interval. When samples were concentrated within groups and dispersed between groups, QC samples were tightly clustered, emphasizing that the above experimental method is reliable. In addition, the reliability of the model was further validated with the OPLS-DA tools (Figures 3A, B, D, E). Subsequent results demonstrated that the samples were all within the 95% confidence interval, indicating that the study model had good predictability and repeatability with no overfitting. The classification of 1,374 metabolites identified in this experiment comprised 478 lipids and lipid-like molecules, 209 organoheterocyclic, 208 phenylpropanoids and polyketides, 140 organic acids and derivatives, 114 benzenoids, 93 organic oxygen compounds, and others (Figure 2C).

The screening conditions for the differential metabolites in the current study were VIP greater than 1 and *P*-value less than 0.05. As illustrated in Figure 2D, the characteristic differential metabolites of the three groups compared (gygc vs. qmgc, qmgc vs. qmlc, and gylc vs. qmlc) were 78, 86, and 132, respectively, suggesting that the samples underwent very active chemical reactions in each of three conditions, and the picking time and processing would have an impact on the quality of “Hefeng tea.”

### Quality differences between gygc and qmgc from DEMs

In the volcano plot of 592 differential metabolites of gygc and qmgc (Figure 3C), top 15 differential metabolites with up- and down-regulation folds can be adopted as marker compounds for the identification of qmgc and gygc (Figure 3G). It is noteworthy that the chemical classification of 30 DEMs was diverse, which established that the leaves-picking time was responded to multiple metabolic pathways. Six flavonoids exhibited a noticeable alteration with the up-regulation of luteolin 7-glucoside, maysin, theadibenzotropolone A, in addition to the down-regulation of comosin, daidzein, and egonol glucoside. Meanwhile, three terpenoids significantly altered the up-regulation of alpha-dihydroartemisinin, an artemisinin that treats malaria. Terpene volatiles has been previously identified as the main contributors to floral aroma, and are further known to influence the sensory quality of green tea (12). Furthermore, enilconazole, a fungicide widely used in agriculture, especially in the cultivation of citrus fruits, was previously documented to be markedly reduced in gygc (13).

The significant variations between the 30 DEMs provided an initial insight into the differences between the two green teas. Figure 2B further illustrates the mechanism how the DEMs (which included catechins, anthocyanins, theaflavin, phenolic acids, etc.) determine green tea quality.

#### Catechins

Catechins are a type of phenolic compounds very abundant in green tea and have a bitter and astringent taste. In our study, among the five monomeric catechins, namely catechin (C), epicatechin (EC), epigallocatechin (EGC), and gallocatechin (GC), were up-regulated, while epigallocatechin gallate (EGCG) was down-regulated. Accumulating works have shared similar conclusions with our study (5). For instance, young leaves possess more catechins compared to mature leaves. Moreover, a high level of catechins was previously associated with the strong floral aroma of “Xinyang Maojian.” Meanwhile, the longer daylight and increased light intensity from Qingming Festival to Guyu Festival (15 days) are known to promote catechin content in tea leaves, whereas gallocatechin, epicatechin gallate, and catechin gallate content in tea plant healing tissues decreases. Furthermore, catechin and epicatechin also contribute to the sweet aftertaste of green tea (14).

#### Anthocyanins

Additionally, we found that the relative content of anthocyanins, prodelphinidin A1, prodelphinidin B, delphinidin 3-glucoside, and 3,3′-di-O-galloylprocyanidin B5 were all significantly down-regulated in gygc. This was in contrast to previous studies, wherein the levels of proanthocyanidin A1, prodelphinidin A1, and prodelphinidin A2 3′-gallate were much higher in late spring tea compared to early spring tea (15). The latter could be attributed to the fact that our samples were collected from high-altitude rather than low-altitude areas. Nevertheless, some of our findings are in accordance with previous reports (16). The high-grade “Huangshan Maofeng” exhibited more proanthocyanidins than the low-grade kind. Existing reports suggest that the accumulation of anthocyanins gave the leaves a purple color, and also greatly enhances the bitterness of green tea (17). We speculate that for qmgc, the dark greenish oily color and bright green soup may be attributed to the high anthocyanin content.

#### Theaflavin

Theaflavin is responsible for the yellowing of the tea broth, and further contribute to the astringency and aftertaste of tea leaves (18). Our findings revealed that gygc presented with up-regulation of isoneotheaflavin 3-O-gallate, theaflavin, in addition to down-regulation of quinone. Meanwhile, quinone is an intermediate product in the oxidation of catechins to theaflavin, while we learned that the tea broth of gylc was yellowish, presumably the catechins were being converted from quinone to theaflavin at that time.

#### Phenolic acids

Gallic acid, ellagic acid, and chlorogenic acid are essential for the synthesis of flavonols and catechins in tea plants. In our study, we found that the acids of gallic, chlorogenic, and ellagic were all present at higher levels in qmgc. Gallic acid is further associated with astringency, sourness, and sweet aftertaste of green tea (19), while young and tender parts normally possess higher gallic acid content especially in early spring teas (7, 15). Chlorogenic acid has also been identified as a flavor modifier for high-quality products and to improve sensory quality (20). Furthermore, ellagic acid is regarded as a marker of high-quality white tea (21).

#### Alkaloids

Major well-known alkaloids in tea include caffeine, theobromine, and theophylline, of which caffeine is the most dominant alkaloid in tea, accounting for more than 90% of the total alkaloids and the primary source of bitterness in green tea (22). Surprisingly, it has been uncovered that caffeine is not the cause of the bitterness difference between qmlc and gylc since statistical analysis revealed that caffeine was not present in the DEMs of gylc vs. qmlc. This particular finding is inconsistent with one previous report (15), wherein higher levels of caffeine were documented in the young parts of tea plants. In addition, theobromine and theophylline were up-regulated and down-regulated respectively.

#### Flavonols and flavonol glycosides

Flavonols and flavonol glycosides are astringent compounds and serve to enhance the bitterness in green tea (14). The relative contents of kaempferol, kaempferol 3-alpha-L-arabinofuranoside, myricetin, and myricetin 3-neohesperidoside were found to be markedly up-regulated in gygc. Fermentation process helps reduce the content of kaempferol-o-glucosides, leading to astringency reduction (22). One previous study also found that kaempferol-glucose-rhamnose-glucose in low-grade green tea was present at a much higher concentration than in the high-grade kind (16).

#### Free amino acids

Free amino acids that are involved in the formation of aroma substances impart green tea with a refreshing taste, such that their presence can be an indicator of tea quality (23). L-theanine is one such amino acid in tea and is known to decrease in content with shoot maturation (15). In our study, we uncovered that the content of L-theanine remained at a stable level. Meanwhile, the up-regulation of L-phenylalanine is regarded as the precursor for flavonoid synthesis, which eventually produces catechins, anthocyanins, and flavonol glycosides. In addition, L-phenylalanine can augment the astringency and bitterness (18), but also positively correlated with the freshness of green tea (24). Additionally, in our study, more freshness was detected when the content of L-glutamic acid was up-regulated in qmgc. This is in accordance with a previous study in which higher levels of L-glutamic acid were noted in early spring tea than late spring tea (7). On the other hand, when L-targinine was down-regulated in gygc, there was a decrease in the bitterness of tea broth (25).

#### Soluble sugars

Additional experimentation in our study revealed that the contents of D-maltose, neokestose, and phlorin were all down-regulated in gygc. These soluble sugars have a sweet taste, and have also previously been shown to be effective in alleviating the bitterness of tea broths (14). A similar study found that early spring tea accumulated a large number of sugars and sugar alcohols, which contribute to an increase in the quality of green tea (26). Herein, we hypothesized that the decrease in soluble sugar of gygc was caused by unfolded leaves. In that case, net photosynthesis was negative and soluble sugars were heavily consumed for cellular energy supply leading to decreased soluble sugar of gygc. Furthermore, the sensory analysis support that the sweetness was more pronounced in qmgc.

#### Organic acids

Organic acids including caffeoylmalic acid, limonexic acid, and jasmonic acid were all down-regulated in gygc, and further contributed to the sour and fruity flavors of qmgc, which is much in accordance with a recent study (7). Moreover, our findings also pointed out that the accumulation of organic acids in early spring tea could effectively resist the invasion of pathogenic bacteria and diminish the use of pesticides to improve the quality of tea leaves. Meanwhile, abscisic acid, an important phytohormone, was significantly up-regulated, which affected the quality of green tea by acting on lipid and flavonoid metabolism (27).

#### Other compounds

The contents of methyl jasmonate and jasmolone were all found to be down-regulated in gygc. Methyl jasmonate is a phytohormone involved in plant defense under adversity conditions, and possesses a floral, creamy aroma (23). Meanwhile, Jasmolone is associated with the enhanced sweetness of green tea (1).

Furthermore, the contents of benzaldehyde and 3,4-dihydroxybenzaldehyde were both diminished in gygc. Normally, these compounds are associated with a specific almond odor in tea products (28). Benzaldehyde has also been previously shown to create a distinct herbal aroma (29).

The content of chlorophyll b was found to be up-regulated in gygc, and further contributed significantly to the transition from light green to yellow-green color in qmlc and gylc. Chlorophyll b represents a yellow-green photosynthetic pigment and its up-regulation is associated with enhanced photosynthesis in tea plants.

The above findings highlighted that qmgc and gygc possess varying metabolomic profiles, and the quality differences between them were determined by a variety of complex interactions of compounds including catechins, free amino acids, alkaloids, and flavanols. In addition, the metabolic profile of qmgc described high-quality green tea with light green soup, prominent fresh flavor, sweet aftertaste, and low bitterness and astringency, which was highly consistent with the sensory evaluation of qmgc and gygc. However, in our study, the pattern of metabolite variation from the samples collected at different picking times did not exactly match the previous Low altitude area research.

### Metabolic differences between gylc and qmlc

The processing of green tea must be fried at high temperatures and the chemical reactions are mediated non-enzymatically (23). Therefore, it is helpful to understand the quality differences of corresponding commercial green tea by discussing the differential metabolites and key metabolic pathways of gylc and qmlc. As illustrated in Figures 3F, H, a total of 30 significant DEMs were directly related to green tea quality such as flavonoids, alkaloids, and amino acids. Among them, flavonoids and alkaloids usually aid plants adapt to their environment, and large amounts of secondary metabolites may be detected from samples grown in an abnormal environment which adds certain stressors to the plants.

Furthermore, we found that picrotin was down-regulated and its content was positively correlated with altitude. The reason may be attributed to the increased temperatures during rainy season, and that tea plants have not to produce large amounts of picrotin to combat low-temperature stress.

Additionally, previous studies have shown that gallocatechin and oolongtheanin, a dimeric catechin formed from epigallocatechin and epigallocatechin gallate with a bitter taste, are present in much higher levels in gylc (30). Moreover, we found that the content of prodelphinidin A1 was significantly lower in gylc, which is in accordance with the works of Zeng et al. (7).

Interestingly, the contents of some compounds that functioned as antibiotics had significant changes. Capsaidiol is a natural fungicide found in peppers formed *via* the isoprenoid pathway from 5-epi-aristolochene, and exerts an antibacterial effect on *Helicobacter pylori* (31). Moracin M is a phytoantitoxin isolated from *Morus alba* infected *Fusarium solani* (32). In addition, enilconazole and capsaidiol were down-regulated in gylc, whereas moracin M was up-regulated. These changes attracted the notice of the symbiotic microorganisms which appear to respond to the timing of fresh tea picking. Additional analysis of the DEMs of gylc and qmlc (Supplementary Table 1) revealed that lycoperdic acid and citrinin were up-regulated, while nivalenol and validamycin B were down-regulated in gylc. The synthesis of lycoperdic acid imparts a pungent odor and antibacterial properties to the sporophore of *Lycoperdon perlatum* (33). The study performed by Bryla et al. found that Nivalenol produced by *Fusarium graminearum* (34) was down-regulated greatly. Meanwhile, validamycin B, an agricultural antibiotic isolated from *Streptomyces hygroscopicus* (35), has also been shown to be down-regulated. On the other hand, citrinin, a fungal toxin with a wide range of biological activities *in vitro* (36) was up-regulated.

As illustrated in Figure 3I, a comprehensive analysis (including enrichment analysis and topological analysis) for the pathways of DEMs was performed by MetPA. Five key pathways in qmlc and gylc were screened with a high correlation to DEMs as follows: flavone and flavonol biosynthesis (*p* = 0.044592, impact = 0.8), isoquinoline alkaloid biosynthesis (*p* = 0.44741, impact = 0.5), phenylalanine metabolism (*p* = 0.54683, impact = 0.5), cysteine and methionine metabolism (*p* = 0.63443, impact = 0.19231), and tyrosine metabolism (*p* = 0.83274, impact = 0.27273). These key metabolic pathways involved the synthesis of flavonoids, alkaloids, and amino acids of tea plants and the biosynthesis of flavonoid, phenylpropanoids, flavone, flavonol, tyrosine, and tryptophan, which were important pathways in early and late spring teas (7), especially flavone and flavonol biosynthesis serve as a key pathway to differentiate tea quality from early, mid and late spring timing (8). From the above findings, the metabolic profiles provided some insight into the relevant physiological properties of fresh tea leaves and the foundation to select the best pick timing.

### Transcriptomic information of gylc and qmlc

To further elucidate the molecular mechanisms underlying the quality differences between gygc and qmgc, RNA-Seq analysis was utilized to investigate the differences between gylc and qmlc. In the high-quality data (Supplementary Table 2) obtained from transcriptome sequencing, the raw database and valid database were 43.04 G and 42.06 G, while the accuracy of sequencing Q20 and Q30 were 99.96 and 97.64%. The clean reads were compared with the genome of “Shuchazao” and the valid data covered 87.94% of the reference genome, indicating that the transcriptome data were reliable and could be used for subsequent analyses.

**FIGURE 4:**
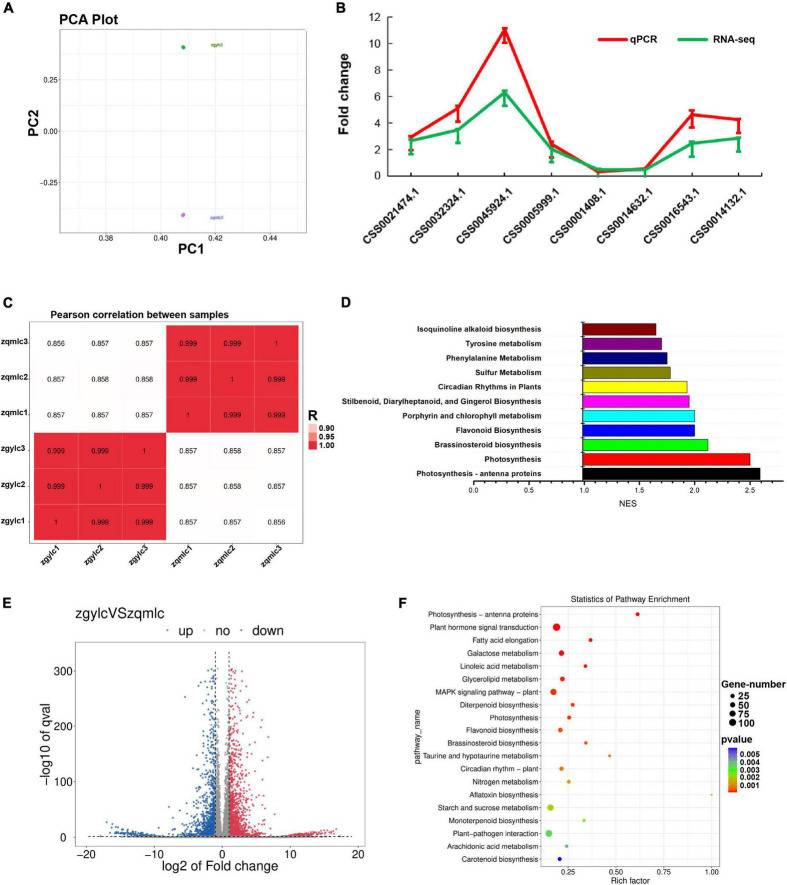
Results of transcriptomic analysis of gylc vs. qmlc. **(A)** PCA plots of qmlc and gylc transcriptomic analyses. **(B)** Results of qPCR validation of transcriptomic data. **(C)** Pearson correlation coefficient plot of zgylc vs. zqmlc. **(D)** GSEA enrichment results for zgylc vs. zqmlc. **(E)** Gene expression volcano plot of zgylc vs. zqmlc. **(F)** KEGG enrichment results of zgylc vs. zqmlc.

Results of PCA analysis, which could prove the biological replicates of qmlc and gylc, indicated that gene expression was closely related to the phenotypic changes in the samples (Figure 4A). From the Pearson correlation coefficient plot, the correlation of the within-group samples was higher than that of the between-group samples (Figure 4C). For the differential gene expression analysis (| log2FC| ≥ 1 and *q* < 0.05 as the criteria), a total of 4,018 genes were considered differentially expressed and of which 2,327 genes were up-regulated, while 1,791 genes were down-regulated (Figure 4E). To validate the reliability of the RNA-Seq results, eight DEGs related to flavonoid metabolism were utilized for RT-qPCR analysis (Figure 4B, Supplementary Figures 1, 2, and Supplementary Table 3) and the results of which revealed that the expression patterns of these genes were highly consistent with those of RNA-Seq analysis.

According to the differential gene KEGG enrichment analysis (Figure 4F), a large number of pathways associated with tea plant growth and development were enriched, which was in perfect agreement with the phenotypes of qmlc and gylc (Figure 1). From Qingming to Guyu Festival (15 days), sunlight duration became longer and tea plants responded to circadian rhythm—plant. Moreover, the activation of the MAPK signaling pathway helped tea leaves with spreading and greening. Plant hormone signal transduction and brassinosteroid biosynthesis provided hormones for the growth of tea shoots. Photosynthesis, photosynthesis-antenna proteins, and carotenoid biosynthesis are further known to enhance photosynthesis activity in young leaves. Some researchers (37) have suggested that the gradual increase of chlorophyll is involved in carbon fixation, and the photosynthesis would differ from the accumulation of flavor metabolites and the tea quality. Meanwhile, enhanced photosynthesis is also known to facilitate the accumulation of free amino acids and aroma components in tea leaves (6). Besides, previous studies have shown that the biosynthesis of flavonoid, diterpenoid, and monoterpenoid, which is closely related to secondary metabolism and flavor quality of tea leaves, were enriched. In addition, pathways related to plant disease resistance, such as plant-pathogen interaction, aflatoxin biosynthesis were also strengthened, which highlighted that the effect of endophytic bacteria on the quality of gygc and qmgc cannot be ignored.

Since KEGG enrichment analyses results were conflicting with the data from DEMs. GSEA (gene set enrichment analysis) was utilized to compensate for the lack of effective information mining of micro-effective genes by traditional enrichment analysis. The screening criteria for GSEA analysis were | NES| > 1, NOM *p*-val < 0.05, and FDR *q*-val < 0.25. Subsequent results (Figure 4D) showed that flavonoid biosynthesis (| NES| = 2, NOM *p*-val = 0.00, FDR *q*-val = 0.00), isoquinoline alkaloid biosynthesis (| NES| = 1.65, NOM *p*-val = 0.014, FDR *q*-val = 0.184), tyrosine metabolism (| NES| = 1.73, NOM *p*-val = 0.00, FDR *q*-val = 0.00) and phenylalanine metabolism (| NES| = 1.75, NOM *p*-val = 0.00, FDR *q*-val = 0.00), all of which were significantly enriched. Together, these findings highlighted the metabolic pathways (phenylalanine metabolism and flavone, flavonol biosynthesis) as the key pathways for the quality difference of gylc and qmlc.

Herein, for further exploring more about the key metabolism relative to quality differences of qmgc and gygc, the transcriptomic and metabolomic data of the samples were combined. Subsequently, correlations were calculated by randomly selecting differential genes and differential metabolites using the “spearman” algorithm. As shown by the heat map (Supplementary Figure 3), the differential metabolites of catechin, procyanidin, and gallic acid were strongly associated with cinnamic acid hydroxylase (C4H), flavonol synthase (FLS), and hydroxycinnamoyltransferase (HCT).

**FIGURE 5:**
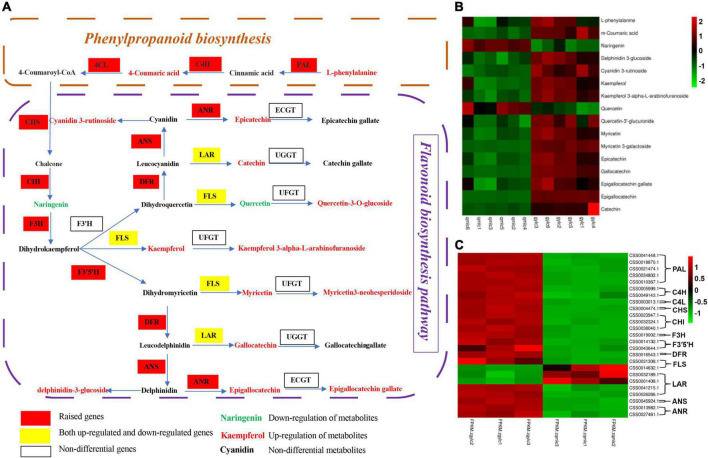
The key metabolic pathways of gylc and qmlc. **(A)** Diagram of the key metabolic pathways of qmlc and gylc. PAL, phenylalanine ammonia-lyase; C4H, cinnamic acid hydroxylase; 4CL, coumadin CoA ligase; CHS, chalcone synthase; CHI, chalcone isomerase; F3H, flavonoid 3-hydroxylase; F3′H, flavonoid 3′-hydroxylase; F3′5′H, flavonoid 3′ 5′-hydroxylase; DRF, dihydroflavonol 4-reductase; LAR, leucoanthocyanidin reductase; UFGT, UDP glucose-flavonoid 3-o-glcosyl-transferase; ANR: anthocyanidin reductase; ANS: anthocyanidin synthase; FLS: flavonol synthase. **(B)** Key metabolic pathways involve DEMs. **(C)** Key metabolic pathways involve DEGs.

Additionally, Figure 5 illustrates the DEMs and DEGs changes in phenylalanine metabolism, flavone, and flavonol biosynthesis, and it can be figured out that the biosynthesis of flavonoids begins with phenylalanine. With the participation of phenylalanine ammonialyase (PAL), cinnamic acid hydroxylase (C4H), and coumadin CoA ligase (4CL), phenylalanine, there was a production of the important intermediate 4-Coumaroyl-CoA which provides the precursor for the subsequent biosynthesis of flavonoids. Phenylalanine and all eight DEGs encoding PAL, C4H, and 4CL were significantly up-regulated in gylc, and it can be speculated that this may contribute to the increase in flavonoid abundance of gylc. PAL has also been reported to serve as the key enzyme for catalyzing the production of cinnamic acid from phenylalanine and its expression corresponds to the catechin content in tea plants (38). 4-Coumaroyl-CoA produced naringenin in the presence of chalcone synthase (CHS) and chalcone isomerase (CHI). Naringenin is a known stable intermediate in flavone and flavonol biosynthesis, and naringenin provides the basic carbon skeleton for flavonoid synthesis (39). Naringenin was down-regulated in gylc and all four DEGs encoding CHS and CHI were up-regulated, which indicated gene expression had opposite trending to the metabolites. The reason may be the increased subsequent flavonoid synthesis led to naringenin depletion.

Naringenin would further produce dihydrokaempferol with the action of flavonoid 3-hydroxylase (F3H), while dihydrokaempferol produced three flavonols along three separate pathways. The flavonoid 3′-hydroxylase (F3′H) and flavonoid 3′ 5′-hydroxylase (F3′5′H) catalyze the biosynthesis of dihydroquercetin and dihydromyricetin respectively. In addition, they further produced quercetin, kaempferol, and myricetin in the presence of flavonol synthase (FLS). These three flavonols would transfer into flavonol glycosides with the action of UDP glucose-flavonoid 3-o-glcosyl-transferase (UFGT). When kaempferol was up-regulated, its flavonol glycosides kaempferol 3-alpha-L-arabinofuranoside and kaempferol 3-O-alpha-L-rhamnofuranoside were down-regulated. On the other hand, when quercetin was down-regulated, its flavonol glycoside quercetin-3′-glucuronide was up-regulated and when myricetin was up-regulated, its flavonol glycoside myricetin 3-galactoside was up-regulated. From the encoding genes, there were no significant changes in the F3′H encoding gene. Genes of *CSS0014132.1* and *CSS0043644.1* encoding F3′5′H were up-regulated and *CSS0048887.1* was down-regulated. Genes of *CSS0045924.1* and *CSS0031308.1* encoding FLS were up-regulated but *CSS0014632.1* was down-regulated. Meanwhile, there were no significant expression differences in the UFGT encoding genes.

Dihydroquercetin and dihydromyricetin form colorless anthocyanins in the presence of dihydroflavonol 4-reductase (DFR), and subsequently, produce colored anthocyanins by anthocyanidin synthase (ANS) catalysis and are finally converted to stable anthocyanins. Cyanidin 3-(6″-p-coumarylsambubioside) and delphinidin 3-glucoside were up-regulated and Cyanidin 3-(6-feruloylglucoside) 5-(6-malonylglucoside) was down-regulated in our study. Moreover, the genes of *CSS0016543.1* encoding DFR and *CSS0045924.1* encoding ANS were significantly up-regulated.

Colorless anthocyanins can be directly reduced to non-epi-type catechins C and GC by leucoanthocyanidinreductase (LAR). Additionally, colorless anthocyanins can be successively catalyzed by the anthocyanidinsynthase (ANS) and anthocyanidinreductase (ANR) to produce the phenotypic catechins EC and EGC. These catechins can be further synthesized into ester catechins by the action of UDG-galloyl-1-o-β-D-glucosetransferase (UGGT) and EC-1-O-galloyl-B-D-gallicacyltransferase (ECGT). Herein, the genes of *CSS0032189.1* and *CSS0001408.1* encoding LAR were down-regulated and *CSS0041215.1* and *CSS0026266.1* were up-regulated. Additionally, the genes of *CSS0013982.1* and *CSS0027461.1* encoding ANR were up-regulated. Moreover, five catechins including catechin (C), epicatechin (EC), epigallocatechin (EGC), gallocatechin (GC), and epigallocatechin gallate (EGCG) all up-regulated, indicating the up-regulated genes exerted a dominant role in the differential genes of LAR. There were no significant expression differences between the genes encoding UGGT and ECGT.

Through flow charts, key pathways directly related to gylc and qmlc quality were regulated by DEGs. Our work lays the foundation for future research into the relationships and molecular mechanisms of secondary metabolite accumulation in tea. However, there may be more complex regulatory mechanisms explaining this phenomenon, and the same requires further elaboration in future studies.

### Endophytic bacteria of gygc and qmgc and their effects on the tea quality

Both metabolomic and transcriptomic data from tea samples suggest that the quality differences between gygc and qmgc may be attributed to symbiotic microorganisms. Endophytic bacteria are a special class that can colonize healthy plant tissues for a long time, and further establish a harmonious association with the plant in symbiotic relationships. This hypothesis was determined by high-throughput sequencing of endophytic bacteria (qm16s and gy16s) from gylc and qmlc. Species richness, evenness, and sequencing depth of gy16s and qm16s were evaluated by alpha diversity analysis (Supplementary Table 4). Our findings revealed that goods coverage was 1 for all samples, indicating high completeness of sequencing data and all bacteria in the samples could be detected. Chao1 and observed species were significantly lowered for gy16s, which meant that the bacterial diversity of gy16s expanded.

**FIGURE 6:**
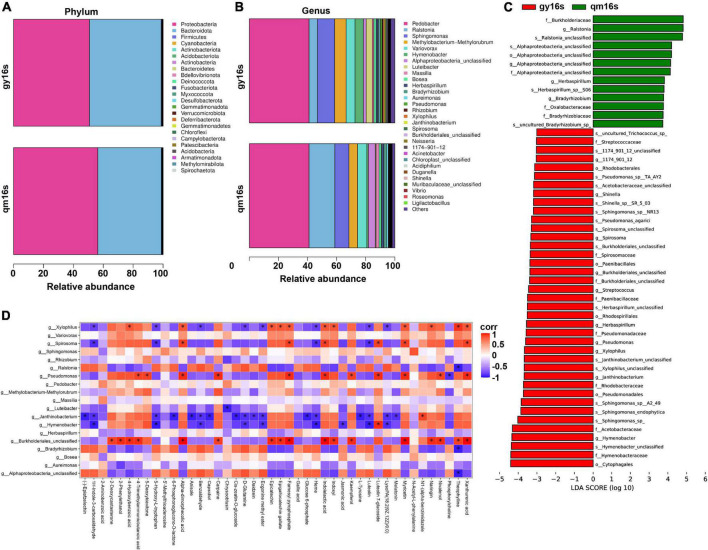
Symbiotic bacterial diversity analysis of qmlc and gylc. **(A)** Differences between gy16s vs. qm16s at the phylum level. **(B)** Differences between gy16s vs. qm16s at the genus level. **(C)** Results of EfSe analysis of gy16s vs. qm16s. **(D)** Heat map of association analysis of DEMs and commensal bacteria for gylc and qmlc. *P < 0.05.

As illustrated in Figures 6A, B, the diversity of the samples was very low at the phylum level and *proteobacteria* and *bacteroidota* were the dominant species. At the genus level, gy16s exhibited a more complex colony structure. Apart from a significant down-regulation of the relative abundance of ralstonia and an up-regulation of the relative abundance of spirosoma, the composition and proportions of dominant species were similar in both samples, which suggested that the two samples shared a core bacterial flora. Surprisingly, ralstonia was reported to be the causal agent of cyanobacteria in plants (40). Moreover, infection of tea plants can lead to poor quality tea and cause economic losses. The inappropriate use of synthetic chemicals to control pests and diseases has become a major problem in the tea industry (41). For this reason, tea samples from the plants without any human intervention were focused on for follow-up observations. Until 26 July 2022, there were no signs of disease in these tea plants. As tea plants on Wufeng mountain were grown for 15 years without pesticides or chemical fertilizers intervention and even the metabolomic data did not contain any evidence of chemical pesticides, the symbiotic microorganisms, the tea plants and its surrounding have produced a stable and harmonious ecosystem. The mechanism by which tea plants infected with the cyanobacteria produce “self-healing” warrants further exploration, especially given that this mechanism could be developed into a novel type of pesticide to improve tea quality by reducing the use of traditional pesticides.

EfSe analysis serves as a tool for the discovery and interpretation of biomarkers for high-dimensional data, and functions as a combination of non-parametric testing and linear discriminant analysis. As illustrated in Figure 6C, the relative abundances of most bacteria were up-regulated in gy16s. Similar to the results in Figure 6B, *Ralstonia*, the pathogen of bacterial wilt, was found to be down-regulated at the genus and species levels. Similarly, *Alphaproteobacteria* was observed to be down-regulated in order, family, genus, and species, and also serves other many functions like phosphate solubilization, IAA production, siderophore production, and ammonia production (42). The relative abundance of s__*Herbaspirillum*_sp__S06, g__*Herbaspirillum* was also down-regulated. *Herbaspirillum* sp., a class of nitrogen-fixing bacteria, and further reported to enhance the effective use of selenate and selenite by tea plants and promote the growth of branch lateral shoots after pruning (43). The relative abundance of s__*Sphingomonas*_sp, s__*Sphingomonas_endophytica*, and s__*Sphingomonas* sp. A2-49 was significantly up-regulated in gy16s. *Sphingomonas* is a well-known common bacterium in tea gardens (44). o__*Pseudomonadales* were significantly up-regulated and their representative strain *Pseudomonas* sp. strain GN6 exerts the functions of phosphate solubilization, IAA production, siderophore production, and ammonia production (45). s__*Janthinobacterium*_unclassified and g__*Janthinobacterium* are significantly up-regulated, such that *Janthinobacterium* has been reported to possess the ability to resist fungi such as *Alternaria brassicicola* (46). Furthermore, J*anthinobacterium* has previously reported as being a coldness-resistant and low-nutrient-needed bacterium, and these characteristics facilitate tea plants to adapt alpine plantations and form a stable colonization (47).

According to correlation analysis between DEMs of gylc and qmlc and endophytic bacteria (Figure 6D), *Burkholderiales* and *Pseudomonas* exhibited positive correlations with DEMs, whereas there were negative correlations between *Janthinobacterium* and *Hymenobacter* with DEMs. Meanwhile, Epigallocatechin gallate, epicatechin were significantly positively correlated with *Xylophilus* and *Burkholderiales.* Catechin and proanthocyanidin biosynthesis have been reported to activate poplar defense against *Melampsora laricipopulina* (48). Additional experimentation revealed that theophylline was negatively correlated with *Alphaproteobacteria, Ralstonia*, but positively correlated with *Xylophilus, Burkholderiales*. Additionally, benzaldehyde was negatively correlated with *Janthinobacterium, Hymenobacter*, and *Xylophilus*. Myricetin was-positively correlated with *Burkholderiales, Pseudomonas, Spirosoma*, and *Xylophilus*. Kaempferol was positively correlated with *Burkholderiales* and *Pseudomonas*. Jasmonic acid was negatively correlated with *Hymenobacter*. D-Glutamine and L-Tyrosine were negatively correlated with *Janthinobacterium*. All these findings collectively suggested that the dynamics of the symbiotic bacterial communities were closely associated with DEMs that determined the quality of qmgc and gygc. Nivalenol was positively correlated with *Pseudomonas* and *Burkholderiales*, therefore it can be inferred that the synthesis of nivalenol might come from these two bacteria and the symbiotic microbes protect tea plants from pathogens by synthesizing antibiotics.

Briefly speaking, the endophytic bacteria inside fresh tea leaves can directly or indirectly influence the quality of Hefeng tea. These bacteria tend to colonize healthy tea plant tissues for a long time and establish a harmonious symbiotic environment. In addition to being directly associated with the DEMs, endophytic bacteria can also regulate the growth rate of tea plants by fixing nitrogen and synthesizing phytohormones, which in turn improve metabolites related to tea quality. From a certain point of view, endophytic bacteria address the root problem of pesticide residues by protecting tea plants from pathogens and reducing pesticides and fertilizers.

## Conclusion

The pick timing of green tea leaves exerts a detrimental effect on their sensory quality from broth color, aroma, and flavor. At a molecular level, the metabolites including catechins, free amino acids, alkaloids, flavonols, soluble sugars, and organic acids vary with the pick timing, leading to teas with different aromas and flavors. In our findings, the variation pattern of differential metabolites associated with green tea quality at different picking times did not exactly match the results of previous studies carried out at lower altitudes. The combined analysis of metabolomic and transcriptomic from fresh tea samples highlighted that flavonoid biosynthesis and phenylalanine metabolism serve as the key pathways responsible for the quality differences of gygc and qmgc. In this pathway, the content of catechins, flavonols, and anthocyanins was regulated by the expression of DEGs and exhibited an up-regulation, which in turn enhanced the bitterness of gygc.

On the other hand, both transcriptomic and metabolomic analysis data on the tea samples revealed that endophytic bacteria indirectly promote tea quality by strengthening plants defense ability and augmenting their growth. Additionally, the microbial diversity analysis verified that endophytic bacteria directly correlate DEMs to influence tea product quality. Metabolites were significantly altered in tea products pre- and post-processing.

Previous studies tend to test the differential metabolites of fresh tea leaves or the differential metabolites of processed tea leaves to analyze the tea quality, which was insufficient. Our study sought to provide a novel theoretical basis for how different pick timing affects the abundance and mechanism of tea metabolites for subsequent research. This study also guided high altitude area (e.g., Hefeng) green tea to find the optimal solution between quality and economic benefits.

## Data availability statement

The original contributions presented in this study are included in the article/Supplementary material, further inquiries can be directed to the corresponding author.

## Author contributions

HX: methodology, investigation, software, data curation, and writing—original draft. JY and YX: software and data curation. HZ: funding acquisition, supervision, methodology, and writing—review and editing. All authors contributed to the article and approved the submitted version.

## Footnotes

1
https://www.cnki.com.cn/Article/CJFDTotal-SPZH201222038.htm

## Supplementary material

Supplementary Figure 1 Melting curve.

Supplementary Figure 2 Amplification curve.

Supplementary Figure 3 Results of association analysis of DEMs and DEGs for qylc and qmlc. **P* < 0.05.
