# Supplementary figures and images for: The molecular mechanisms of quality difference for Alpine Qingming green tea and Guyu green tea by integrating multi-omics
Source: Front Nutr. 2023 Jan 6;9:1079325. doi: 10.3389/fnut.2022.1079325 (PMC9854344; doi:10.3389/fnut.2022.1079325)

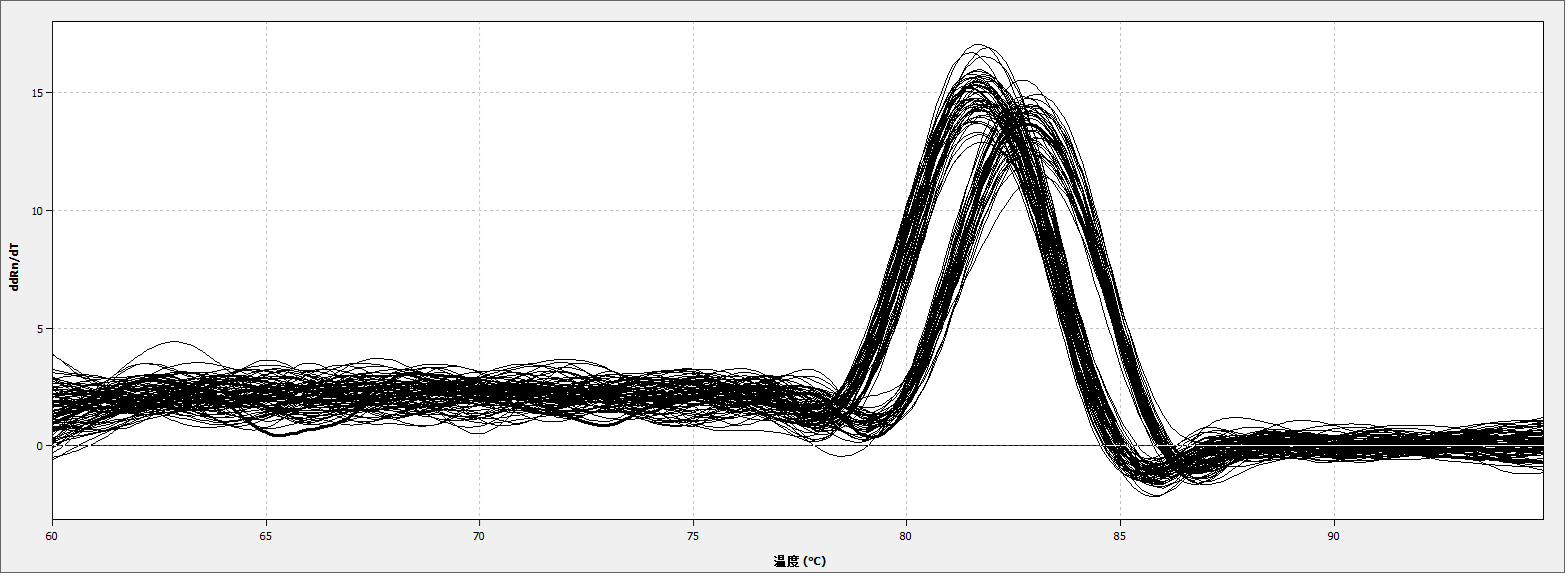

Supplement: Supplementary Figure 1 — Melting curve. [file Image_1.tiff]

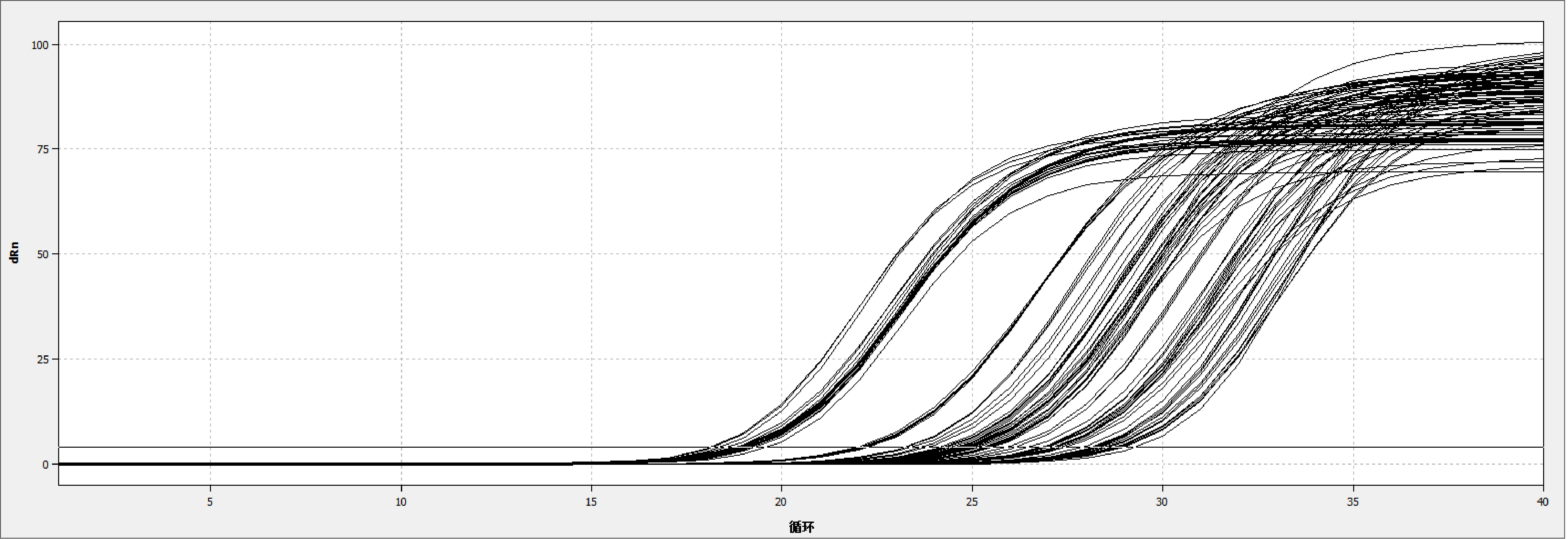

Supplement: Supplementary Figure 2 — Amplification curve. [file Image_2.tiff]

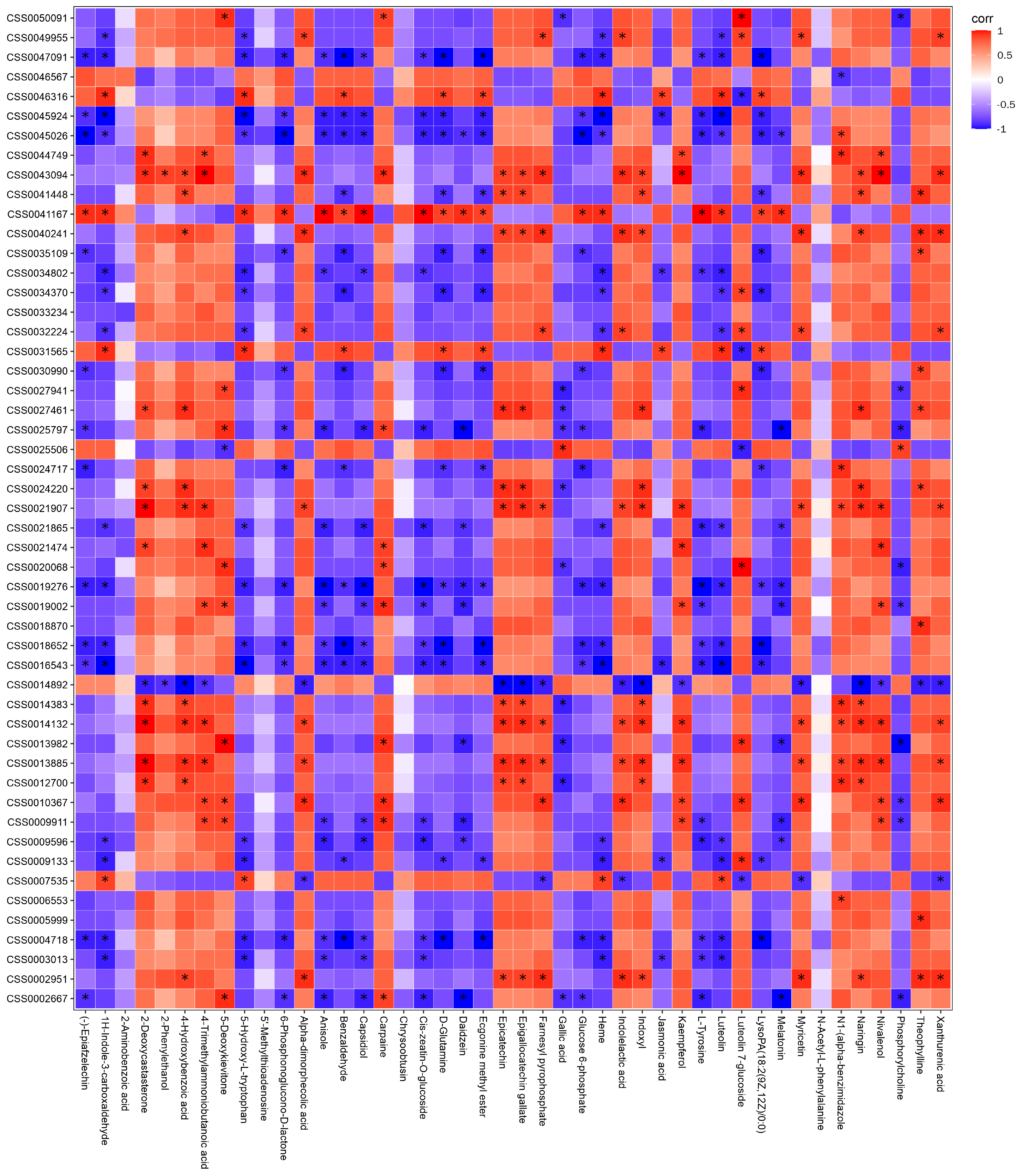

Supplement: Supplementary Figure 3 — Results of association analysis of DEMs and DEGs for qylc and qmlc. *P < 0.05. [file Image_3.tiff]
