# Supplementary material for: The molecular mechanisms of quality difference for Alpine Qingming green tea and Guyu green tea by integrating multi-omics
Source: Front Nutr. 2023 Jan 6;9:1079325. doi: 10.3389/fnut.2022.1079325 (PMC9854344; doi:10.3389/fnut.2022.1079325)
Supplement: Supplementary file 4 [file Table_1.docx]

**Tab. 1** **Sensory quality scores of green teas**

| **Samples** | **Appearance (25%)** | **Aroma**  **(25%)** | **Taste**  **(30%)** | **Brew color**  **(10%)** | **Infused leaves**  **(10%)** | **Total score** |
| --- | --- | --- | --- | --- | --- | --- |
| gygc | 88±2.12a | 95.2±2.16a | 88.2±3.11b | 88.8±2.58b | 82.4±2.50b | 89.38±2.51a |
| qmgc | 97.8±2.28b | 89.4±2.70b | 94.6±2.96a | 94.6±2.40a | 97.8±2.16a | 94.42±2.60b |
