# Supplementary material for: The molecular mechanisms of quality difference for Alpine Qingming green tea and Guyu green tea by integrating multi-omics
Source: Front Nutr. 2023 Jan 6;9:1079325. doi: 10.3389/fnut.2022.1079325 (PMC9854344; doi:10.3389/fnut.2022.1079325)
Supplement: Supplementary file 5 [file Table_2.doc]

**Tab. S2 Valid transcriptomic data statistics for samples.**

| **Sample** | **Raw Data Read** | **Raw Data Base** | **Valid Data Read** | **Valid Data Base** | **Valid Ratio(reads)** | **Q20%** | **Q30%** | **GC content%** | **exon** | **intron** | **intergenic** |
| --- | --- | --- | --- | --- | --- | --- | --- | --- | --- | --- | --- |
| zgylc1 | 50703090 | 7.61G | 49498136 | 7.42G | 97.62 | 99.96 | 97.64 | 43.50 | 87.30 | 6.12 | 6.58 |
| zgylc2 | 50271862 | 7.54G | 49072444 | 7.36G | 97.61 | 99.96 | 97.69 | 43.50 | 87.08 | 6.27 | 6.65 |
| zgylc3 | 52513092 | 7.88G | 51246116 | 7.69G | 97.59 | 99.96 | 97.61 | 43.50 | 86.88 | 6.41 | 6.70 |
| zqmlc1 | 43518004 | 6.53G | 42633938 | 6.40G | 97.97 | 99.96 | 97.51 | 43.50 | 88.58 | 5.03 | 6.39 |
| zqmlc2 | 44927792 | 6.74G | 44002762 | 6.60G | 97.94 | 99.97 | 97.69 | 43.50 | 88.46 | 5.10 | 6.44 |
| zqmlc3 | 44925868 | 6.74G | 43952360 | 6.59G | 97.83 | 99.96 | 97.61 | 43.50 | 87.94 | 5.46 | 6.60 |
