# Supplementary material for: The molecular mechanisms of quality difference for Alpine Qingming green tea and Guyu green tea by integrating multi-omics
Source: Front Nutr. 2023 Jan 6;9:1079325. doi: 10.3389/fnut.2022.1079325 (PMC9854344; doi:10.3389/fnut.2022.1079325)
Supplement: Supplementary file 6 [file Table_3.doc]

**Tab. S3 Primers for qPCR validation**

| **primer** | **sequence** | **PCR product（bp）** |
| --- | --- | --- |
| GAPDH-F | AGCAAGGACTGGAGAGGTGGAAG | 139 |
| GAPDH-R | CGACAGTGGGAACACGGAAAGC |
| 21474.1-F | GTCGGTTCTGGTCTCGCTTCTATG | 149 |
| 21474.1-R | CCTGGATGGTGCTTCAATTTGTGTG |
| 5999.1-F | CTCGCCAAAGAAGTCCTCCACAC | 136 |
| 5999.1-R | TCCTCATCTTCCTCCAGTGCTCAC |
| 32324.1-F | GATGGTGAGATGGGGAAAGCTGATG | 142 |
| 32324.1-R | TCCTCAACCCAACCGCATTCAAC |
| 14132.1-F | CAGGGTTCGGAGTCCAATGAGTTC | 136 |
| 14132.1-R | GACGCTTCATTTCCCTCTCAATCCC |
| 16543.1-F | CGCAGAAGATAACGGCCCTG | 149 |
| 16543.1-R | GTCCTTGGGTCAGCATTGGG |
| 14632.1-F | AGAAGGCGGTGGTGTCGAGATC | 132 |
| 14632.1-R | CTCCCTCCATTTCCAACTTCAACCC |
| 1408.1-F | CCTCGTGGATGCTCTAAAACTGGTC | 129 |
| 1408.1-R | CGCTTGATGTTTCCGGCTTCTTTG |
| 45924.1-F | CAGCATACCAGCCGAGTACATTAGG | 147 |
| 45924.1-R | CCAGCCTCCTCCACCATCCTC |
