# Supplementary material for: The molecular mechanisms of quality difference for Alpine Qingming green tea and Guyu green tea by integrating multi-omics
Source: Front Nutr. 2023 Jan 6;9:1079325. doi: 10.3389/fnut.2022.1079325 (PMC9854344; doi:10.3389/fnut.2022.1079325)
Supplement: Supplementary file 7 [file Table_4.doc]

**Tab. S4 Statistics of valid data for 16s sequencing.**

|  | **Quality control** | | | | | | | | | **Alpha** | | | | | |
| --- | --- | --- | --- | --- | --- | --- | --- | --- | --- | --- | --- | --- | --- | --- | --- |
| **Sample** | | **Raw_Tags** | **Raw_Bases** | **Valid_Tags** | **Valid_Bases** | **Valid%** | **Q20%** | **Q30%** | **GC%** | **observed_otus** | **shannon** | **simpson** | **chao1** | **goods_coverage** | **pieloue** |
| qm16s3 | | 80657 | 40.33M | 70132 | 29.26M | 86.95 | 95.66 | 89.12 | 52.43 | 145 | 4.12 | 0.88 | 145.00 | 1.00 | 0.57 |
| qm16s2 | | 87080 | 43.54M | 77941 | 32.48M | 89.51 | 95.92 | 89.57 | 51.82 | 206 | 3.92 | 0.88 | 206.09 | 1.00 | 0.51 |
| qm16s1 | | 83464 | 41.73M | 74494 | 31.29M | 89.25 | 95.69 | 89.06 | 50.85 | 149 | 3.21 | 0.77 | 149.00 | 1.00 | 0.44 |
| gy16s3 | | 85996 | 43.00M | 74427 | 31.12M | 86.55 | 95.65 | 89.07 | 51.61 | 486 | 5.07 | 0.87 | 486.08 | 1.00 | 0.57 |
| gy16s2 | | 87514 | 43.76M | 76288 | 31.76M | 87.17 | 95.72 | 89.19 | 51.67 | 339 | 4.54 | 0.88 | 339.33 | 1.00 | 0.54 |
| gy16s1 | | 85051 | 42.53M | 75604 | 31.64M | 88.89 | 95.97 | 89.60 | 52.07 | 377 | 5.21 | 0.94 | 377.27 | 1.00 | 0.61 |
